# Hybrid male sterility between *Drosophila willistoni* species is caused by male failure to transfer sperm during copulation

**DOI:** 10.1186/s12862-015-0355-8

**Published:** 2015-05-01

**Authors:** Alberto Civetta, Chelsea Gaudreau

**Affiliations:** Department of Biology, University of Winnipeg, Winnipeg, Canada

**Keywords:** Hybrid male sterility, *Drosophila*, Speciation, Sperm transfer

## Abstract

**Background:**

The biological concept of species stresses the importance of understanding what mechanisms maintain species reproductively isolated from each other. Often such mechanisms are divided into premating and postmating, with the latest being the result of either prezygotic or postzygotic isolation barriers. *Drosophila willistoni quechua* and *Drosophila willistoni willistoni* are two subspecies that experience reproductive isolation. When a *D. w. quechua* female is crossed with a *D. w. willistoni* male, the hybrid males (F_1QW_) are unable to father progeny; however, the reciprocal cross produces fertile hybrids. Thus, the mechanism of isolation is unidirectional hybrid male sterility. However, the sterile F_1QW_ males contain large amounts of motile sperm. Here we explore whether pre-copulatory or post-copulatory pre-zygotic mechanisms serve as major deterrents in the ability of F_1QW_ males to father progeny.

**Results:**

Comparisons of parental and hybrid males copulation durations showed no significant reduction in copulation duration of F_1QW_ males. Interrupted copulations of the parental species confirmed that sperm transfer occurs before the minimum copulation duration registered for F_1QW_ males. However, we found that when females mate with F_1QW_ males, sperm is not present inside the female storage organs and that the lack of sperm in storage is due to failure to transfer sperm rather than spillage or active sperm dumping by females.

**Conclusions:**

Sterility of F_1QW_ hybrid males is primarily driven by their inability to transfer sperm during copulation.

**Electronic supplementary material:**

The online version of this article (doi:10.1186/s12862-015-0355-8) contains supplementary material, which is available to authorized users.

## Background

Speciation is the process by which new species are formed throughout the course of evolution. Among sexually reproducing organisms, speciation is frequently the result of either physical or biological barriers that prevent interbreeding [1]. Biological barriers to interspecies hybridization can occur in any form of male - female incompatibilities prior to or after the initiation of copulation. Pre-copulatory isolation may be caused by the physical inability to copulate or by behavioral rejection. Premating behavioral isolation can arise as a consequence of females avoiding male mating signals or male rejection of heterospecific females [2-4]. In addition, the duration of copulation may be considered an isolating mechanism whereby a shortened copulation prevents the successful transfer of sperm. For example, when *Drosophila simulans* females mate with *Drosophila sechellia* males, the copulation duration is shorter than in conspecific mattings and very few sperm is transferred [5]. Even under conditions in which copulation duration is long enough to achieve transfer of sperm and seminal products, postmating prezygotic mechanisms (PMPZ) can interfere with fertilization.

A myriad of studies have documented and investigated the mechanistic basis of PMPZ. One of the first and most striking examples came from species of the cricket genus *Allonemobius*, where competitive PMPZ in the form of conspecific sperm precedence (CSP) and non-competitive gametic isolation driven by the inability of heterospecific males to induce females to lay eggs, can solely prevent gene flow among species [6-8]. Both competitive and non-competitive PMPZ barriers have been also studied among closely related species of *Drosophila*. For example, among females singly-mated to heterospcific males, sperm dumping by females and poor sperm storage have been reported to negatively affect the fate of heterospecific sperm [9,10]. The use of transgenic males with fluorescently labeled sperm has recently shown a common set of sperm precedence mechanisms in competitive settings with a major role of active sperm displacement of resident heterospecific sperm by incoming conspecific sperm and female control of sperm utilization through sperm ejection and fertilization bias [11].

One of the most common forms of reproductive isolation reported between species is hybrid male sterility [12]. Hybrid male sterility is identified by the inability of interspecies hybrids to father progeny and it is commonly linked to sperm problems or even complete lack of motile sperm [13-18]. Hybrid males are often classified as fertile even if they produce a single motile sperm, but sperm motility does not necessarily ensures fertility. This is because sperm that is normal both in size and structure and appears to be able to properly move could still suffer from subtle physiological defects. Hybrid males from crosses between a *Drosophila willistoni willistoni* female with a *Drosophila willistoni quechua* male (F_1WQ_) are fully fertile with normal sperm motility and produce large numbers of offspring. When a *D. w. willistoni* male is crossed with a *D. w. quechua* female, the hybrid males (F_1QW_) are unable to father any progeny [18,19]. However, we have recently found that the sterile hybrid males have normal amounts of motile sperm [18]. This suggests that either subtle sperm problems impede these males from properly fertilizing eggs or that an earlier problem, during or shortly after copulation, might effectively render these males sterile.

Here we test how precopulatory and postcopulatory pre-zygotic mechanisms influence the sterility of the F_1QW_ hybrid males by using a combination of copulation duration, interrupted copulation and sperm tracking assays. We find that the sterile male hybrids are unable to transfer sperm to females. Our results do not rule out the possibility that the motile sperm is not able to fertilize eggs (sterility *sensu stricto*), but instead identify an earlier postcopulatory prezygotic problem as the main factor effectively causing sterility. Overall the finding illustrates the complexity of interspecies isolation barriers and stresses the need to properly phenotype hybrid defects in order to clearly identify what mechanisms prevent them from fathering progeny.

## Methods

### *Drosophila* Stocks maintenance and handling

*Drosophila* species used in this study were *Drosophila willistoni willistoni* (14030–0811.16) from Laguna Negra, Rocha in Uruguay and *Drosophila willistoni quechua* (14030–0814.10) from Guadeloupe Island, France. Both stocks were obtained from the San Diego *Drosophila* Stock Center (https://stockcenter.ucsd.edu). Flies were reared in bottles containing cornmeal-yeast-agar-molasses (CYAM) medium. Bottles were kept in a 12:12 light–dark cycle and at 21–24 °C. For stock maintenance, flies were allowed to freely mate and laid progeny in fresh media, the adults were discarded after 10 days, and a new generation of newly hatched flies were transferred to fresh media.

Flies used for phenotypic assays or to produce intespecies hybrids were collected by first emptying the bottles from each species stock and collecting newly emerged flies every four hours to ensure virginity. Virgin females and males were separated by sex and placed in vials containing CYAM medium. Flies were maintained at a density of 10–20 flies per vial and aged five to seven days to reach sexual maturity [20] before used in any assay. To produce interspecies hybrids, males and females of different species were mixed together in bottles containing medium and monitored in the same way as parental species stocks prior to collection of progeny for assays.

### Uninterrupted and interrupted matings and sperm storage

Fly pairs, either conspecific or heterospecifics, were aspirated into a food vial to avoid anesthetization. Six different combinations were used, with males and females of the same species or F1 hybrid males with females of either parental species. Three different trials were conducted. First, flies were allowed to complete copulation uninterrupted and copulation duration was recorded. In the next two trials, only parental species were tested and flies were allowed to copulate for either five or nine minutes, then interrupted and separated into vials. After each trial, the female spermatheca and seminal receptacle were dissected using forceps and fine insect pins three hours after copulation. Presence of sperm was assayed using an Olympus inverted microscope with phase contrast optics at 20-40× magnification. Since ovulation in *D. melanogaster* begins approximately one and a half hours after copulation and occurs prior to the full completion of sperm storage [21], dissections were done at three hours to ensure sperm storage in the female would be completed.

### Genitalia

Male external genitalia, and the intromittent organ (aedeagus), from *D. w. willistoni*, *D. w. quechua* and F1 hybrids were dissected in 1xPBS using a Nikon stereoscope. The tissue samples were picked up using a thin brush, placed on dry slides and mounted in mounting medium (IBIDI, Ingersoll, Ontario) under coverslips. Images were captured using a Zeiss Ax10 microscope with a Zeiss AxioCam imaging system. The samples were imaged at a resolution of × 400 magnification.

### Sperm transfer and female dumping

Coverslip cubes were constructed using six 18 x 18 mm coverslips and transparent tape [22]. Fly pairs were lightly anesthetized, placed in a coverslip cube, observed for copulation, and the copulation duration was recorded. Flies that failed to mate within four hours and flies that mated for less than nine minutes were discarded. The cubes were disassembled between one to four hours after the mated flies had been removed, wiped down on the exterior surface and checked for the presence of sperm masses under a phase contrast microscope at 20-40× magnification. The reproductive tract of fully-mated females was dissected in 1× PBS on a microscope slide using forceps within half an hour after mating. The seminal receptacle, spermatheca, and uterus were dissected out from the reproductive tract using fine insect pins. Sperm cells were stained using DAPI and a Zeiss Ax10 microscope was used to check for presence of sperm in any of the storage organs.

### Data analysis

Data was analyzed using One-way analysis of variance (ANOVA) with the nature of the cross as factors. Copulation duration was logarithmically transformed to fit ANOVA assumptions. When significant differences were found among crosses, an *a posteriori* Scheffe’s test was performed to test which cross averages were significantly different from one another. All statistical tests were conducted in SPSS (version 12.0).

## Results

### Copulation duration of parental species and interspecies hybrid males

**Figure 1:**
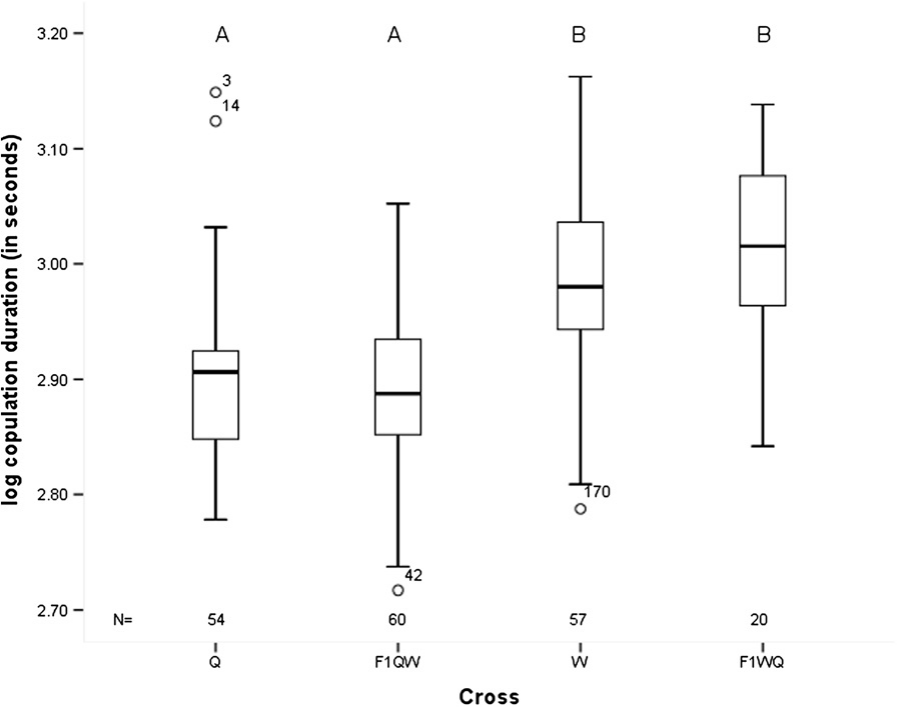
Boxplot showing results from copulation duration assays. Q = *D. w. quechua* females mated to conspecific males; W = *D. w. willistoni* females mated to conspecific males; F1_QW_ and F1_WQ_ = Sterile and fertile hybrid males respectively, mated to both Q and W females. N = Number of flies tested.

Interspecies hybrid males from crosses between *D. willistoni* parental subspecies are either sterile or fully fertile. However, it is unknown whether sterile males suffer any form of reduced copulatory fitness. Copulation duration was measured for the parental species as well as for the interspecies hybrid males mated to both parental females. We found significant differences in copulation duration among parental species and hybrid males backcrossed to parental species females (F_3,187_ = 24.4; P < 0.001) (Additional file 1: Table S1). A Scheffe’s Post-Hoc test identified that F_1QW_ hybrid males mated for the same average amount of time as *D. w. quechua* parents whereas F_1WQ_ hybrid males mated on average for as long as *D. w. willistoni* parents (Figure 1). Given that there were significant differences in copulation duration of the sterile F_1QW_ male hybrids based on whether they mated to *D. w. willistoni* or *D. w. quechua* females (F_1,58_ = 4.27; P = 0.004) (Additional file 1: Table S1), we re-analyzed the data by partitioning the mating types by both females and males involved. We still detected significant differences among mating pairs (F_5,185_ = 15.7; P < 0.001), with the Scheffe’s post-hoc test still grouping F_1WQ_ with *D. w. willistoni* and F_1QW_ with *D. w. quechua* parents. However, F_1QW_ males mated to *D. w. willistoni* females also grouped with *D. w. willistoni* parents (Additional file 1: Table S1). Since copulation duration of F_1QW_ and F_1WQ_ hybrid males is similar to that of the female parent used to produce each particular hybrid, the result suggests that copulation duration is strongly influenced by the X-chromosome origin of the hybrid genome.

### Interrupted copulations and sperm storage in *D. willistoni* species

**Figure 2:**
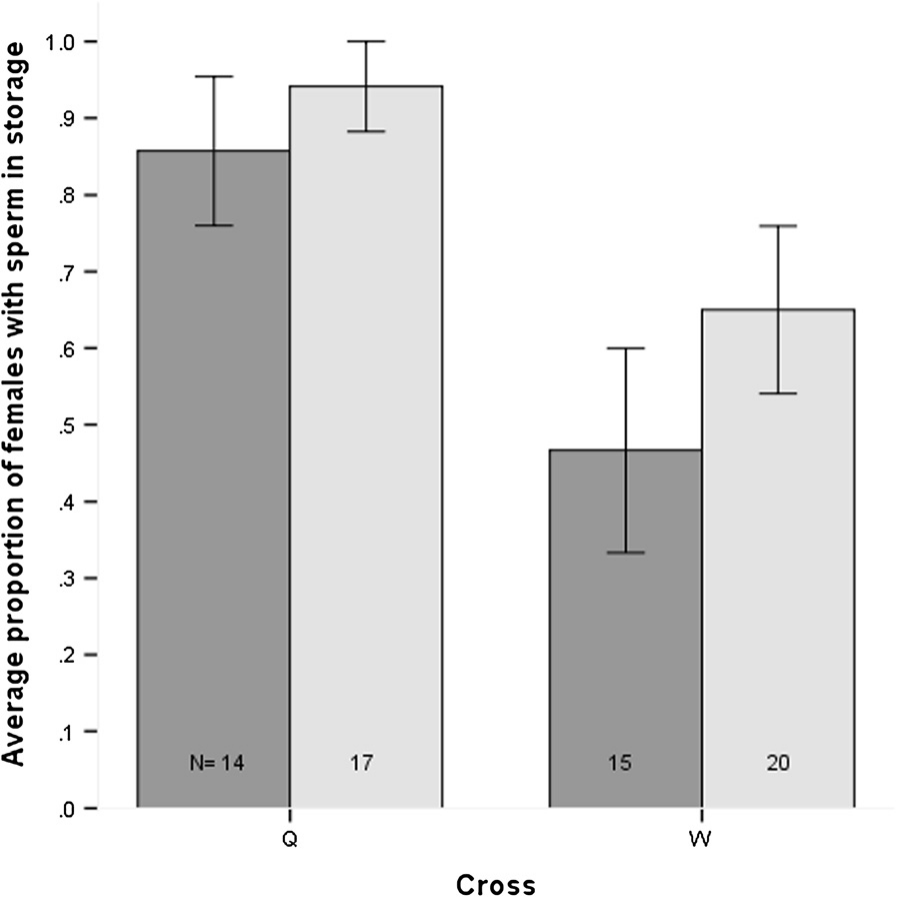
Average proportion of sperm in female storage at 5 (dark grey) and 9 (light grey) minute interrupted copulations for both conspecific *D. w. quechua* (Q) and *D. w. willistoni* (W) matings. Error bars are ± 1 standard error; Numbers within bars (N) are the number of females sampled.

The shortest copulation duration recorded was close to nine minutes (8:41) for a F_1QW_ male mated to a *D. w. quechua* female. Copulation duration can have an impact on the effectiveness of sperm transfer and storage, so we used parental species in interrupted copulation at nine minutes (minimum copulation duration recorded) and at an arbitrary shorter time of five minutes. The data shows that even at five minute interruptions, close to half (47%) of th*e D. w. willistoni* females had sperm in storage, with the proportion increasing to 65% at nine minute interruptions (Figure 2) (Additional file 1: Table S2). This indicates that among fully mated flies, most hybrid males should have sufficient time to transfer and store their sperm inside either of the parental species females.

### Sterile hybrid males fail to transfer sperm

**Figure 3:**
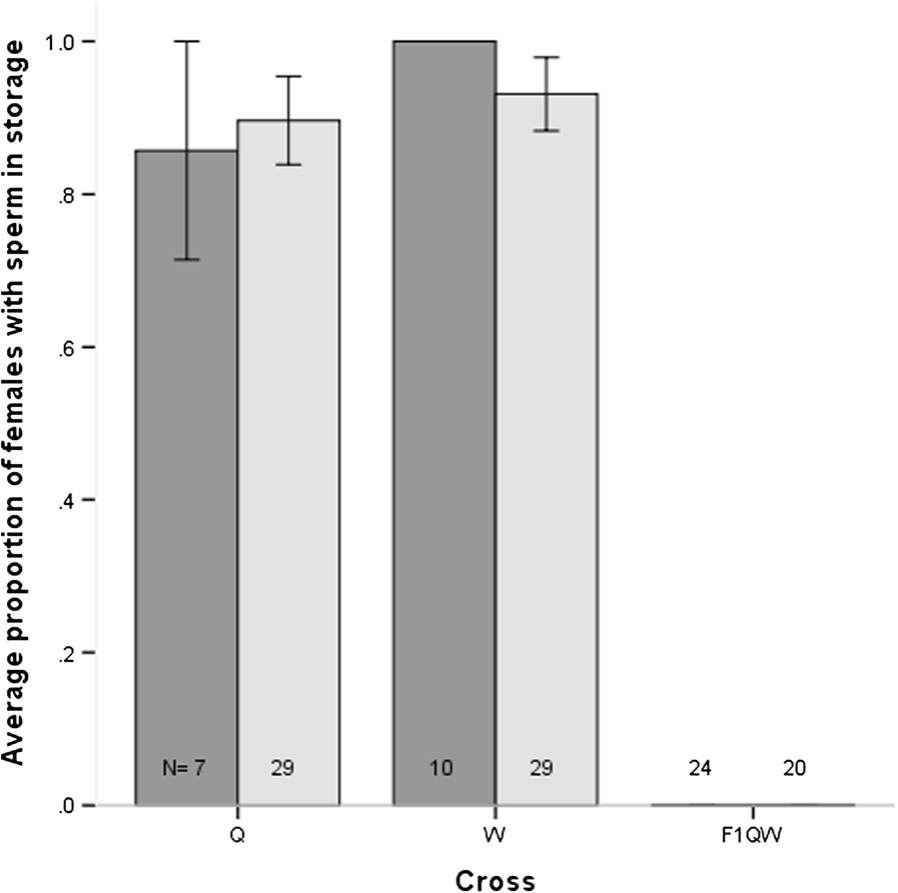
Average proportion of fully mated females with sperm in storage within half an hour (dark grey) and three hours (light grey) after mating. The labels are as in Figures 1 and 2.

While there was not a significant reduction of copulation duration for the F_1QW_ hybrid males with respect to the *D.w. quechua* parents, it is still possible that they either failed to transfer sperm or that there was either passive sperm spillage or active dumping by females. Therefore, female sperm storage organs (bursa, spermatheca and seminal receptacle) were assayed for presence of sperm after allowing males to copulate uninterrupted. No sperm was found inside any storage organ of any female mated to the F_1QW_ males within a half-hour or three hours after mating, while most females mated to parental species had sperm in storage (Figure 3) (Additional file 1: Table S3). The time intervals were chosen based on our knowledge of sperm storage and dumping from *D. melanogatser*. Sperm transfer and storage in *Drosophila melanogaster* starts before copulation is completed and continues to increase rapidly before plateauing at approximately six hours after copulation while dumping of sperm by females can occur between 30 minutes and five hours after copulation [21,22]. Thus, given what we know about *D. melanogaster* and our own observation that sperm can be found in the female storage organs of both *D. w. willistoni* and *D. w. quechua* females mated to conspecific males even within 30 minutes after copulation is completed, we believe that the absence of sperm in the storage organs of females mated to the F_1QW_ males can only be a consequence of either male’s failure to transfer sperm or fast spillage/dumping from the females. Incidentally, a small sample of F_1WQ_ hybrid males was also tested and all females mated to them showed sperm in storage within 30 minutes after the completion of copulation (Additional file 1: Table S3).

**Figure 4:**
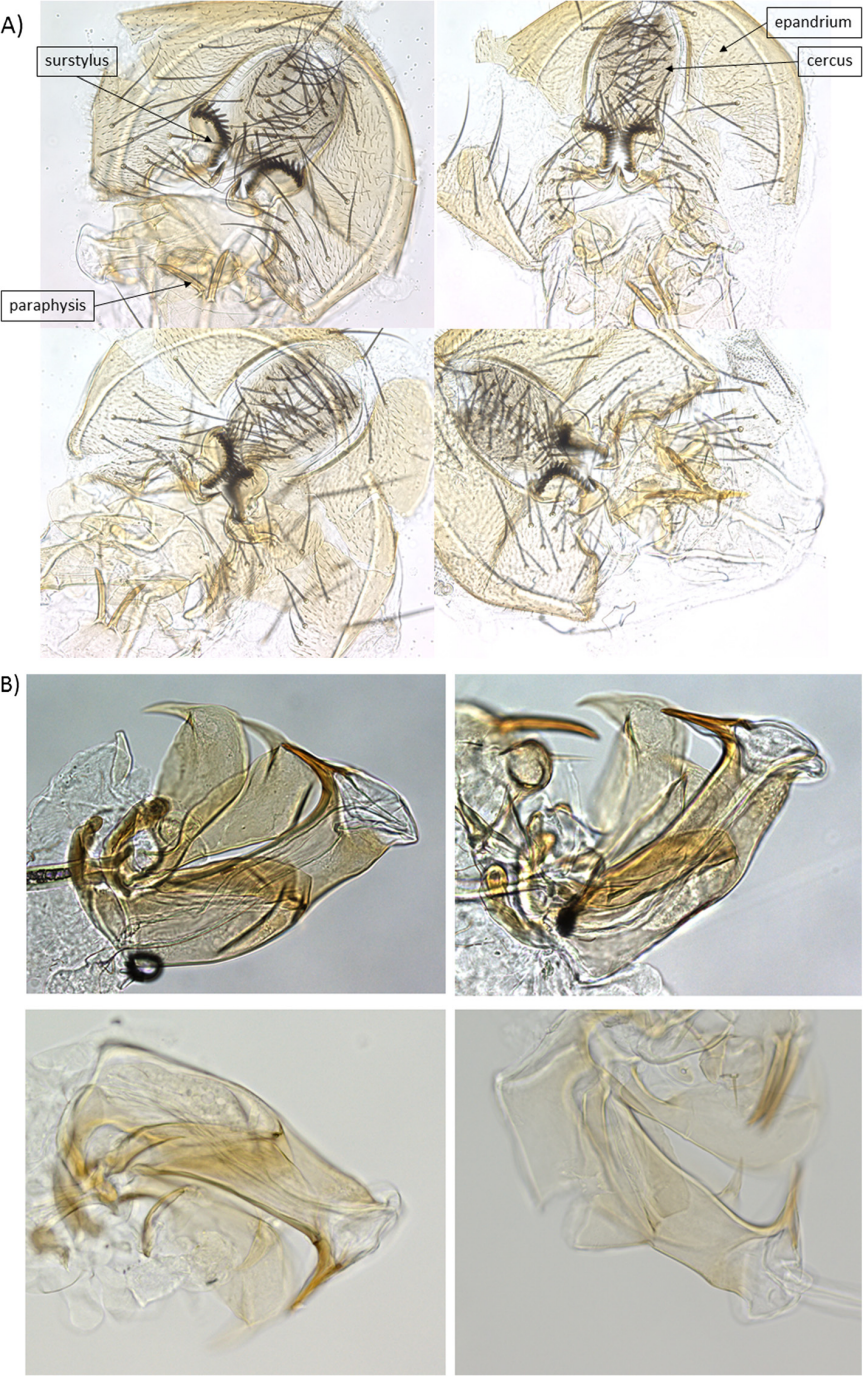
Morphology of the **A**) male external and **B**) male internal (aedeagus) genitalia of *D. w. willistoni*, *D. w. quechua*, F1_WQ_ (fertile) and F1_QW_ (sterile) hybrids are shown clockwise from top left.

Contrary to the morphological differences in the external and internal genitalia of other species of *Drosophila*, the male genitalia showed no evidence of divergence between *D. w. willistoni* and *D. w. quechua* and no clear evidence of atrophy in the hybrids relative to parental species (Figure 4) Thus, it is apparent that the inability of these males to transfer sperm to females is not influenced by major changes in the genitalia of the sterile male.

In order to determine whether the complete lack of any sperm in females that fully copulated with an F_1QW_ male is a consequence of sperm spillage or dumping from their storage organs, we tested parental crosses and F_1QW_ males fully mated to parental females at 30 minutes after copulation for evidence of lost sperm masses. We found no evidence of any sperm masses lost by females (Additional file 1: Table S4). In summary, we found no evidence of any stored sperm in females mated to F_1QW_ males and the females show no evidence of sperm spillage, indicating that F_1QW_ males fail to transfer sperm during copulation.

## Discussion

Speciation can occur instantly, as it is the case for polyploidization in plants, but more frequently it is the result of the action of different isolation barriers that evolve gradually [12]. Thus, it is important to identify how different levels of isolation (e.g. premating, postmating prezygotic and postzygotic) contribute to prevent interbreeding among species pairs. There is also a need to understand the mechanistic basis of the isolating barriers.

In earlier studies of hybrid male sterility, there tends to be a general lack of detailed information on the classification of the “sterility” phenotype. Among species of *Drosophila*, interspecies male hybrids are sometimes classified as fertile based on the presence of motile sperm but such males can be effectively sterile as they are unable to produce progeny [18,23]. In *Drosophila*, sterility is often associated to developmental problems of the male reproductive tract morphology such as atrophy of the seminal vesicles that contain mature individualized sperm or more severe whole or partial testes atrophy [24-26]. Cytological analyses of sterile hybrid males have shown a variety of subtle sperm development changes, such as abnormal sperm individualization timing, excessive cellular debris between sperm bundles or sperm tails with disrupted axoneme to mitochondrial derivatives relationships that can result from anomalies during late stages of sperm development [16,27,28]. Sperm development abnormalities in interspecies sterile hybrids are also common among other species. Interspecies hybrids between species of *Xenopus* produce more undifferentiated spermatids and less sperm with less motility than parental species [29]. House mouse hybrids show a wide range of both meiotic and postmeiotic defects [30,31] and for male hybrids between *M. m. musculus* and *M. m. domesticus* support has been found for chromosomal asynapsis of heterospecific homologous chromosomes during meiosis as a major factor contributing to sterility [32]. There have also been links established between sterility and chromosomal heterogeneity between species of *Drosophila*. For example, in interspecies backcrosses there is a correlation between the amount of heterospecificity among species genomes and sterility [33]. Rapid changes in heterochromatin and hetrochromatin-binding proteins can also contribute to sterility in *Drosophila mauritiana* × *Drosophila simulans* male hybrids [34].

This detailed information on what causes sterility has been valuable but there has been a tendency to directly associate sterility phenotypes to developmental problems affecting the ability of males to produce sperm capable of fertilizing eggs. Thus it is commonly assumed that spermatogenesis genes divergence or misexpression in sterile hybrids might be a key causal contributor or a direct consequence of sterility [35-39]. Our finding of an inability of hybrid males to transfer sperm into females, despite being able to properly engage in copulation for periods of times that should allow the proper transfer of sperm, suggests that sterility is primarily a consequence of a postmating prezygotic problem, but not a spermatogenic defect. The overall morphology of the sterile hybrid male reproductive tract is normal and the males produce motile sperm [18] suggesting that sperm transfer problems are not driven by morphological anomalies. However, the sperm motility does not rule out the possibility that the sperm might be unable to fertilize eggs (i.e. they are sterile *sensu stricto*). Even if sperm defects affect the hybrid males fertilization ability, the primary barrier to fertilization occurs at the sperm transfer stage and prior to any possible sperm fertilization capacity problem. To our knowledge, there have not been reports of complete inability of males to transfer sperm. There is incomplete sperm transfer in crosses involving *D. simulans* females with *D. sechellia* males, but most of these males copulate with heterospecific females for only half the amount of time when compared to a conspecific female [5]. Age can also affect sperm transfer. For example in beetles, younger males transfer less sperm than older males, but the younger males still manage to transfer some sperm [40].

One possible explanation for the failure of sterile hybrid males to transfer sperm is defects in the external or internal genitalia. The external genitalia, particularly the male surstyli and the stout setae are known to serve to clamp *Drosophila* females and in *D. willistoni*, the paraphyses act as a clamp [41]. We found no obvious differences in the morphology of these structures in the sterile F_1QW_ males and an electron microscopy study did not identify *D. willistoni* female structures that could interact with the male structures to facilitate or impede grasping [41]. Variation in the shape of the male aedeagus has been found to be a diagnostic trait among some subspecies of *Drosophila* [42]. Our examination of the *D. willistoni* species and their fertile and sterile male hybrids shows no clear differences in the shape of the aedeagus and no indication of major morphological abnormalities in the sterile males. While a major morphological atrophy was not detected in the F1_QW_ sterile males, a detailed electron microscopy analysis of parental species and hybrids might help identify whether any minor morphological anomaly in the male genital grip during copulation or the intromettent organ impedes proper signalling and contributes to a lack of sperm transfer. It is also possible that defects in the musculature of the genitalia, such as the attached musculature necessary for eversion of the aedeagus, might cause the failure to transfer sperm.

The inability of the sterile hybrid males to transfer sperm could be influenced by genetic factors. Crosses involving *D. w. quechua* females with males of *D. w willistoni* of different geographic origin can occasionally produce fertile male hybrids and differences in chromosomal rearrangements have been identified [19]. However, crosses between *D. w. quechua* females and males from two different strains of *D. w. willistoni* that produce sterile hybrids showed no evidence of fixed differences in chromosome structure between the two species [19]. Alternatively differences in regulation of genes with a role in controlling sperm transfer might contribute to sterility. We have surveyed gene expression of the *D. w. willistoni corazonin* gene ortholog, which is known to affect sperm transfer in *D. melanogaster* [43] but found no evidence of differential gene expression between hybrids and parental species (data not shown). Although *corazonin* shows no signature of expression divergence, most genes within the *Drosophila willistioni* genome are not functionally annotated. Therefore, currently unidentified loci might be functionally important for sperm transfer and could have substantially diverged between these species. With increasing efforts to expand genome resources to other species of *Drosophila*, a genome-wide survey rather than a candidate-gene approach could help identify genes or gene pathways uniquely misregulated in the sterile hybrid. Moreover, mapping the chromosomal location of misregulated genes could help us further explore whether chromosomal structural differences might have in fact contributed to allelic imbalances in expression between the two species.

## Conclusions

One common form of isolation between species is restricted gene flow due to hybrid male sterility. The severity of the sterility phenotype is often measured by counts of sperm motility or the inability of sterile hybrids to father progeny. Hybrid male sterility is often assumed to be related to problems in the ability of males to either produce sperm or spermiogenic defects affecting the sperm capacity to fertilize eggs. Here we establish that hybrid male sterility between *Drosophila willistoni* species is caused by male failure to transfer sperm during copulation. Narrowing down the mechanistic basis of infertility can help direct efforts towards our understanding of speciation and its underlying genetic basis.

## Availability of supporting data

All supporting data is included in the Additional file 1: Tables S1 to Table S4.

## Additional file

Additional file 1: Tables S1, S2, S3 and S4. Copulation duration data, presence of sperm in female storage for interrupted copulations, presence of sperm in storage of fully mated females, and data on female sperm spillage. (One .XLXS file).
